# Physiological β-catenin signaling controls self-renewal networks and generation of stem-like cells from nasopharyngeal carcinoma

**DOI:** 10.1186/1471-2121-14-44

**Published:** 2013-09-27

**Authors:** Yue Cheng, Arthur Kwok Leung Cheung, Josephine Mun Yee Ko, Yee Peng Phoon, Pui Man Chiu, Paulisally Hau Yi Lo, Marian L Waterman, Maria Li Lung

**Affiliations:** 1 Department of Clinical Oncology, Center for Nasopharyngeal Carcinoma Research, University of Hong Kong, L6-02, 21 Sassoon Road, Pokfulam, Hong Kong SAR, China; 2 Department of Microbiology and Molecular Genetics, University of California, Irvine, CA 92697, USA

**Keywords:** Physiological Wnt/β-catenin signaling, Nasopharyngeal carcinoma, Self-renewal network, Chromosome 3 transfer, Stemness transition, Tumor suppressor genes, Cancer stem cell markers

## Abstract

**Background:**

A few reports suggested that low levels of Wnt signaling might drive cell reprogramming, but these studies could not establish a clear relationship between Wnt signaling and self-renewal networks. There are ongoing debates as to whether and how the Wnt/β-catenin signaling is involved in the control of pluripotency gene networks. Additionally, whether physiological β-catenin signaling generates stem-like cells through interactions with other pathways is as yet unclear. The nasopharyngeal carcinoma HONE1 cells have low expression of β-catenin and wild-type expression of *p53*, which provided a possibility to study regulatory mechanism of stemness networks induced by physiological levels of Wnt signaling in these cells.

**Results:**

Introduction of increased β-catenin signaling, haploid expression of β-catenin under control by its natural regulators in transferred chromosome 3, resulted in activation of Wnt/β-catenin networks and dedifferentiation in HONE1 hybrid cell lines, but not in esophageal carcinoma SLMT1 hybrid cells that had high levels of endogenous β-catenin expression. HONE1 hybrid cells displayed stem cell-like properties, including enhancement of CD24^+^ and CD44^+^ populations and generation of spheres that were not observed in parental HONE1 cells. Signaling cascades were detected in HONE1 hybrid cells, including activation of *p53-* and *RB1*-mediated tumor suppressor pathways, up-regulation of *Nanog*-, *Oct4*-, *Sox2*-, and *Klf4*-mediated pluripotency networks, and altered E-cadherin expression in both *in vitro* and *in vivo* assays. qPCR array analyses further revealed interactions of physiological Wnt/β-catenin signaling with other pathways such as epithelial-mesenchymal transition, TGF-β, Activin, BMPR, FGFR2, and LIFR- and IL6ST-mediated cell self-renewal networks. Using β-catenin shRNA inhibitory assays, a dominant role for β-catenin in these cellular network activities was observed. The expression of cell surface markers such as CD9, CD24, CD44, CD90, and CD133 in generated spheres was progressively up-regulated compared to HONE1 hybrid cells. Thirty-four up-regulated components of the Wnt pathway were identified in these spheres.

**Conclusions:**

Wnt/β-catenin signaling regulates self-renewal networks and plays a central role in the control of pluripotency genes, tumor suppressive pathways and expression of cancer stem cell markers. This current study provides a novel platform to investigate the interaction of physiological Wnt/β-catenin signaling with stemness transition networks.

## Background

Multiple groups have shown that different dosage levels of Wnt signaling contribute to distinct cellular activities such as reprogramming, differentiation, tumorigenesis, and epithelial-mesenchymal transition (EMT) events [1-4]. Inappropriate activation of components of this signaling pathway has been observed in some human cancers and differentiating stem cells, in which high levels of Wnt signaling were often detected [1,4-8]. Therefore, Wnt signaling has multiple functions in cell fate determination and is involved in generation of cancer stem cells (CSCs). However, there are unresolved issues for the role of Wnt/β-catenin signaling in the regulation of either self-renewal or differentiation networks in human cells [4,5,7,9,10].

Nasopharyngeal carcinoma (NPC) is a unique cancer, which is particularly prevalent among the southern Chinese, but rare in most other areas around the world [11,12]. Unlike other common tumors, how Wnt signaling influences NPC and crosstalks to other networks to affect cell differentiation and growth, including regulation of CSC markers and EMT events, is unknown. NPC was reported to have an infrequent mutation of tumor suppressor gene (TSG) *p53* and wild-type *RB1* expression [11-14]; they both play critical roles in the control of the reprogramming process, self-renewal, and other cell fate determinations [15-17]. Wnt signaling interacts with p53 signaling [18-20] and usually acts in a dosage-dependent and tissue-specific manner for many cellular processes [1,21-26]. Therefore, it is possible to reveal novel findings by exploring the regulatory mechanism of Wnt signaling in wild-type *p53* expressing tumors such as with NPC HONE1 cells.

We previously established several microcell hybrid cell (MCH) lines derived from HONE1 cells containing a transferred copy of chromosome 3 [11]. Because a physiological or basic level of Wnt signaling acts as a determinant factor in the regulation of stem cells and self-renewing tissues [3,25,27,28] and HONE1 cells have very low endogenous expression of β-catenin, a major mediator of Wnt signaling, we hypothesized that introduction of another copy of the β-catenin gene (*CTNNB1*) via single copy transfer of chromosome 3 may generate physiological levels of β-catenin signaling due to the haploid level of expression of the transferred genes under control by their natural regulators. This approach differs from gene transfection studies that often cause artificial overexpression of the transferred genes [25]. In many cancer cells, stem cell-related genes are often expressed. Exogenous β-catenin signaling may regulate these endogenous signaling networks, thus changing the direction of cellular differentiation. Additionally, *p53*, *RB1,* or other possible TSGs, often serve as negative barriers for the reprogramming and self-renewal processes [15,16]. Delicate control of relevant signaling activities may drive cells into a more de-differentiated status, revealing signaling regulatory mechanisms during the stemness transition process, a series of regulatory relationships that are not fully understood in human cells.

It is important to determine what critical role β-catenin plays in the transferred chromosome by examining the relevant network activities in recipient cells. It is well-accepted now that Wnt/β-catenin signaling interacts with many other signaling networks such as pluripotency, cadherins, EMT, transforming growth factor-β (TGF-β), fibroblast growth factor (FGF), and TSG signaling [1,8,15,16,26,29,30]. If Wnt/β-catenin signaling is activated, these relevant network activities are expected to be detected in treated cells. For example, altered expression of E-cadherin and EMT markers should be found in these cells. Therefore, whether Wnt signaling, initiated at a basic and physiological level, is able to induce other signaling pathways during the progress of stemness transition, or to generate stem-like cells from human cancer cells, such as NPC, is the focus of this study.

## Results

### Monochromosome 3 transfer confers physiological increases of β-catenin that up-regulates expression of core stem cell genes

**Figure 1:**
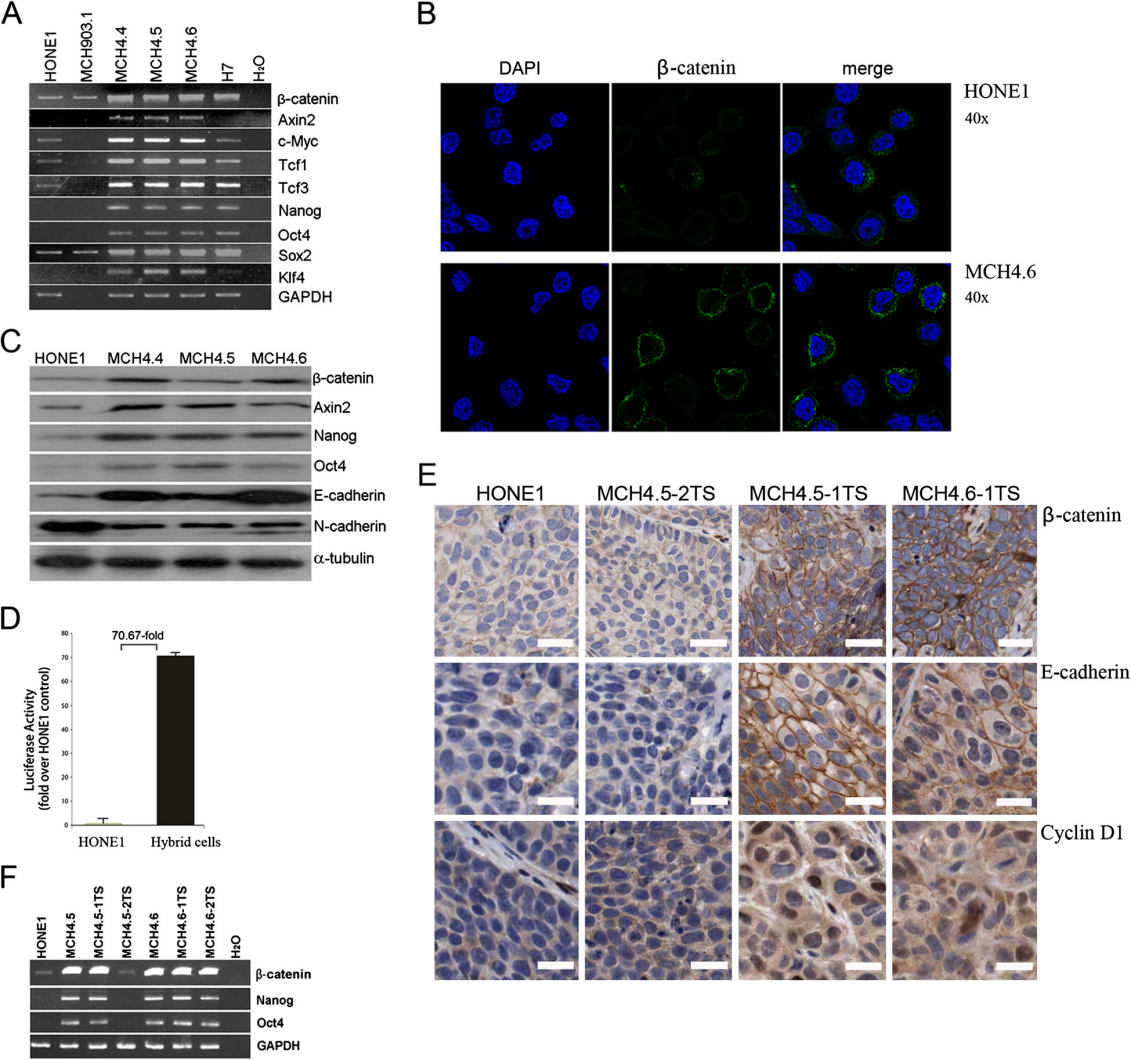
**Exogenous β-catenin signaling induces Wnt pathway and stem cell-related network activities in HONE1 hybrid cells. A**. RT-PCR analyses for HONE1, MCH903.1, HONE1 hybrid cells (MCH4.4/4.5/4.6) and human embryonic stem cells H7. **B**. Immunofluorescence staining shows that β-catenin proteins clearly accumulate in the cellular membrane in most of hybrid cells (MCH4.6). **C**. Western blot analysis reveals that protein expression of β-catenin, Axin2, Nanog, Oct4 and E-cadherin is up-regulated in HONE1 hybrid cells, but N-cadherin is down-regulated. **D**. Luciferase assay shows increased Wnt activities in HONE1 hybrid cells. STOP/SFOP values are increased by 70-fold in MCH4.6 cells compared to parental HONE1 cells. **E**. Immunohistochemical staining shows consistent expression of Wnt target genes in mouse models. In tumor samples, protein expressions of β-catenin, E-cadherin, and Cyclin D1 are strongly up-regulated in tissues derived from two HONE1 hybrid cell lines (MCH4.5-1TS and MCH4.6-1TS), 40x magnification. Bars, 20 μm. **F**. RT-PCR analysis shows that loss of transferred chromosome fragment containing an exogenous β-catenin copy (MCH4.5-2TS) is associated with concomitant loss of up-regulated expression of endogenous genes.

We previously established several HONE1 hybrid cell lines that were confirmed to contain an exogenous copy of the intact chromosome 3, following fusion of parental HONE1 and mouse MCH903.1 donor cells [11]. Figure 1A shows that both HONE1 and MCH903.1 cells have similar and low expression levels of the human β-catenin, consistent with their having physiological levels of β-catenin signaling. Human embryonic stem cells, H7 [31], were used as a positive control for mRNA expression of stem cell genes and β-catenin. The up-regulation of β-catenin expression was clearly detected in all three HONE1 hybrid cell lines, as compared to HONE1, and is similar to that detected in H7 cells. Both *c-Myc* and *Axin2* are major targets of the Wnt pathway and *Tcf1* and *Tcf3* are terminal components of the β-catenin signaling pathway in the nucleus. The expression of *Axin2* was detected in HONE1 hybrid cells, but not in H7 cells and parental HONE1 cells. The expression of *Tcf1, Tcf3,* and *c-Myc* were obviously up-regulated in these HONE1 hybrid cells, compared with parental HONE1 cells (Figure 1A).

Significant expression of pluripotency genes, *Nanog, Oct4*, and *Klf4,* at the RNA level was detected in HONE1 hybrid cells compared to parental HONE1 cells. Both HONE1 and mouse MCH903.1 cells show endogenous expression of human *Sox2* gene, but in HONE1 hybrid cells this gene was up-regulated and had similar expression levels as seen in H7 cells (Figure 1A).

The increased level of β-catenin protein accumulating around membranes was clearly detected in the majority of hybrid cells compared to parental HONE1 cells by immunofluorescence staining (Figure 1B). As expected, up-regulated protein levels of β-catenin, Axin2, Nanog, and Oct4 were detected by Western blot analyses (Figure 1C). Compared with parental HONE1 cells, E-cadherin was up-regulated and N-cadherin was down-regulated in the HONE1 hybrid cells.

### Enhanced Wnt/TCF/LEF network activity is maintained *in vivo*

A luciferase assay was performed using a Wnt/TCF reporter plasmid that carries a large array of Wnt response elements and is, therefore, a sensitive reporter of Wnt signals. As shown in Figure 1D, transient transfection of the superTOPGAL reporter detected an increased level of Wnt signaling by approximately 70-fold in HONE1 hybrid cells.

Immunohistochemical staining of the xenograft tumors demonstrated that β-catenin, E-cadherin*,* and Cyclin D1, the target of Wnt signaling, were strongly up-regulated in tumor segregants (TSs) derived from HONE1 hybrids, as compared with control tumors from the parental HONE1 and MCH4.5-2TS cells. Both β-catenin and E-cadherin proteins clearly accumulated at the membrane (Figure 1E), consistent with the pattern shown in Figure 1B cultured cells.

It has long been known that some regions of transferred chromosomes in TSs are selectively lost during tumor growth and the expression of relevant exogenous genes reverts back to levels similar to parental HONE1 cells [11,12,32-34]. Xenograft tumors derived from HONE1 hybrid cells were, therefore, analyzed for β-catenin expression (Figure 1F). For three of the HONE1 hybrid tumor sets (MCH4.5-1TS/4.6-1TS/4.6-2TS), increased β-catenin levels were evident as well as increased levels of endogenous stem cell genes *Nanog* and *Oct4,* compared to corresponding hybrid cells. A fourth hybrid cell line tumor, MCH4.5-2TS (see Figure 1E), did not show up-regulated expression of exogenous β-catenin and also did not have increased expression of *Nanog* and *Oct4*.

### Physiological expression of exogenous β-catenin is essential for the up-regulation of the endogenous core stem cell network

**Figure 2:**
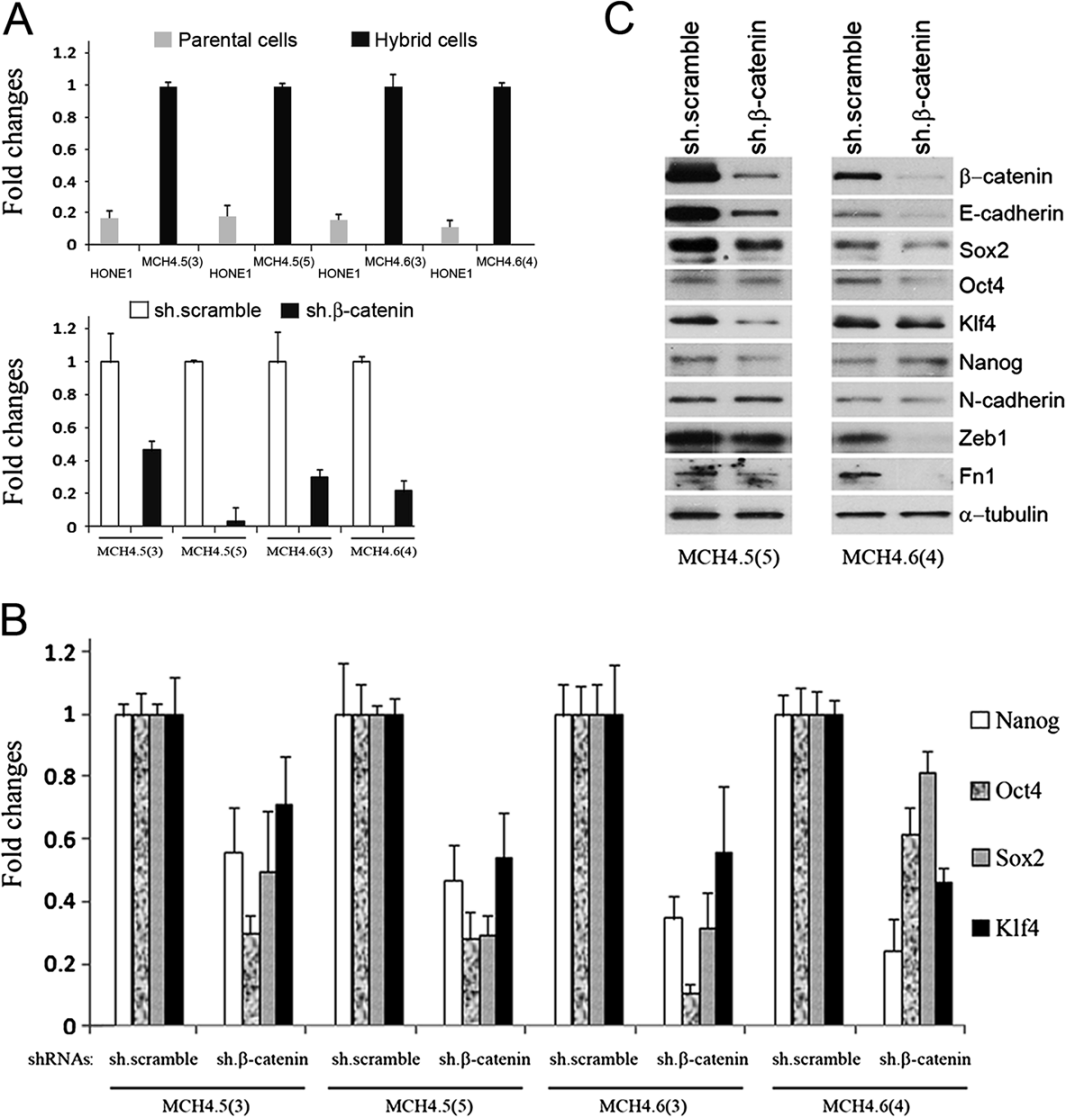
**shRNA knockdown of β-catenin is associated with the regulation of Wnt pathway, EMT, and pluripotency networks. A**. The top panel shows that HONE1 hybrid cells have an up-regulated expression of β-catenin by qPCR analyses, compared to the parental HONE1 cells. MCHs 4.5 and 4.6 are infected by scramble (empty bars) and β-catenin shRNAs (black bars). The number in parenthesis refers to the growth passages of cells after infection. Fold-changes reflect inhibitory expression of β-catenin in these cells compared to corresponding scramble negative controls. β-catenin expression is reduced in all four cell lines, as shown in the bottom panel. **B**. qPCR analyses show inhibited expression of *Nanog, Oct4, Sox2*, and *Klf4* in β-catenin shRNA infected cells. **C**. Western blot assay confirms that β-catenin shRNAs inhibit expression of β-catenin, E-cadherin, core stem cell factors (Oct4, Klf4, and Sox2), and EMT factors (Zeb1 and Fn1) in the treated HONE1 hybrid cells.

To further confirm whether expression of exogenous β-catenin is a major determinant in the regulation of the core stem cell network in HONE1 hybrid cells, we performed inhibitory assays using short hairpin RNAs (shRNAs) that silence β-catenin expression in infected cells. A scrambled shRNA was used as a negative control in all experiments. HONE1 hybrid cells with infected scramble shRNA have up-regulated expression of β-catenin compared to parental HONE1 cell (Top panel, Figure 2A). HONE1 hybrid cells with infected β-catenin shRNA have reduced expression of β-catenin and representative results from various passages of cell populations after infection of shRNA plasmids are shown in the bottom panel, Figure 2A. β-catenin expression was reduced by approximately 54%, 97%, 70%, and 79% in MCH4.5 and MCH4.6 cell lines at different passage levels after infection. In these β-catenin shRNA-infected cells, expression of *Nanog*, *Oct4*, *Sox2*, and *Klf4* was inhibited as observed by qPCR analyses (Figure 2B).

Figure 2C shows that expression of β-catenin, E-cadherin, Sox2, Zeb1, and Fn1 proteins were clearly decreased in both β-catenin shRNA-treated cell lines. The inhibited expression of Oct4 was seen in MCH4.6(4), and inhibition of Klf4 was detected in MCH4.5(5), but no obvious expression change was detected for N-cadherin and Nanog. These results suggest that β-catenin signaling in HONE1 hybrid cells is involved in both stemness and EMT networks.

### HONE1 hybrid cells are poorly differentiated and exhibit stem cell-like properties

**Figure 3:**
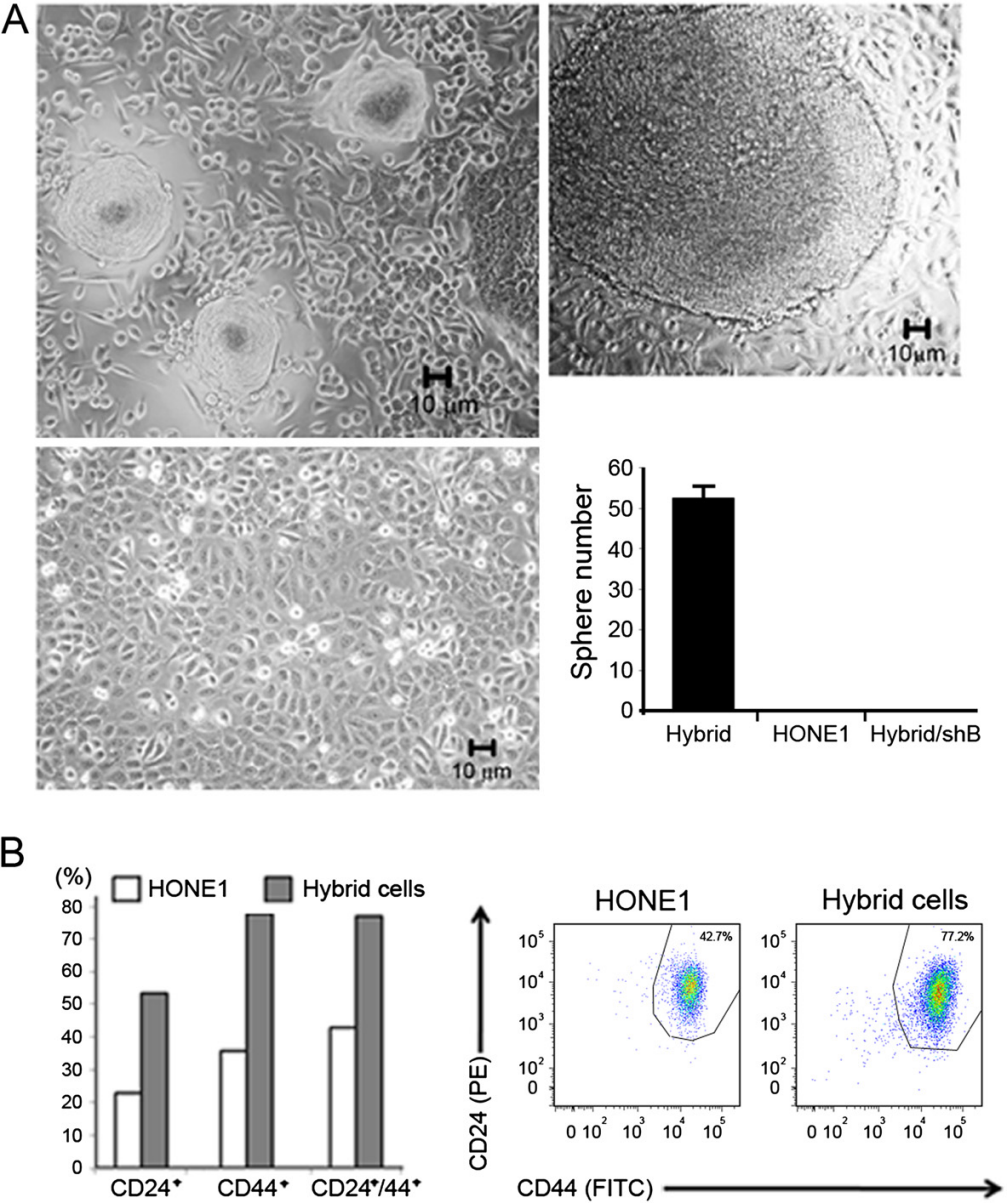
**HONE1 hybrid cells have properties of stem-like cells. A**. Phase-contrast images of spheres that could be grown on the surface of monolayer epithelial cells or bottom of flask (upper right and upper left). The bottom picture shows image of parental HONE1 cell growth. The bar graph indicates that parental HONE1 cells and HONE1 hybrid cells with infected β-catenin shRNA (Hybrid/shB) do not form spheres in the same culture conditions. **B**. FACS analyses (bar chart, Left) show that HONE1 hybrid cells (solid bars) contain more CD24^+^, CD44^+^, CD24^+^/CD44^+^ populations than those of HONE1 cells (empty bars). The figure (right) shows that hybrid cells have increased numbers of double positive CD24^+^/CD44^+^ populations.

Parental HONE1 cells grow as monolayers that do not form spheres under ordinary culture conditions, evidenced by our long-term culture of these cells [11]. In contrast, after 45 to 90 days in standard culture, 158 spheres from independent experiments were observed in the HONE1 hybrid cultures (Figure 3A). The size of all spheres was larger than 20 μM in diameters, which was not detected in parental HONE1 and HONE1 hybrid cell lines with infected β-catenin shRNA. These results suggest that sphere-forming cells had lost contact inhibition and had undergone a dedifferentiation process.

Since CSC markers, CD24 and CD44, are commonly expressed in many stem-like cells, we investigated expression changes of these two markers by FACS analysis. CD24^+^, CD44^+^, and CD24^+^/CD44^+^ populations were markedly increased from 23% to 53.2%, 35.7% to 77.9%, and 42.7% to 77.2%, respectively, in HONE1 hybrid cells compared to parental HONE1 cells (Figure 3B).

### Physiological Wnt levels trigger multiple signaling activities during the regulation of stem cell gene networks

**Figure 4:**
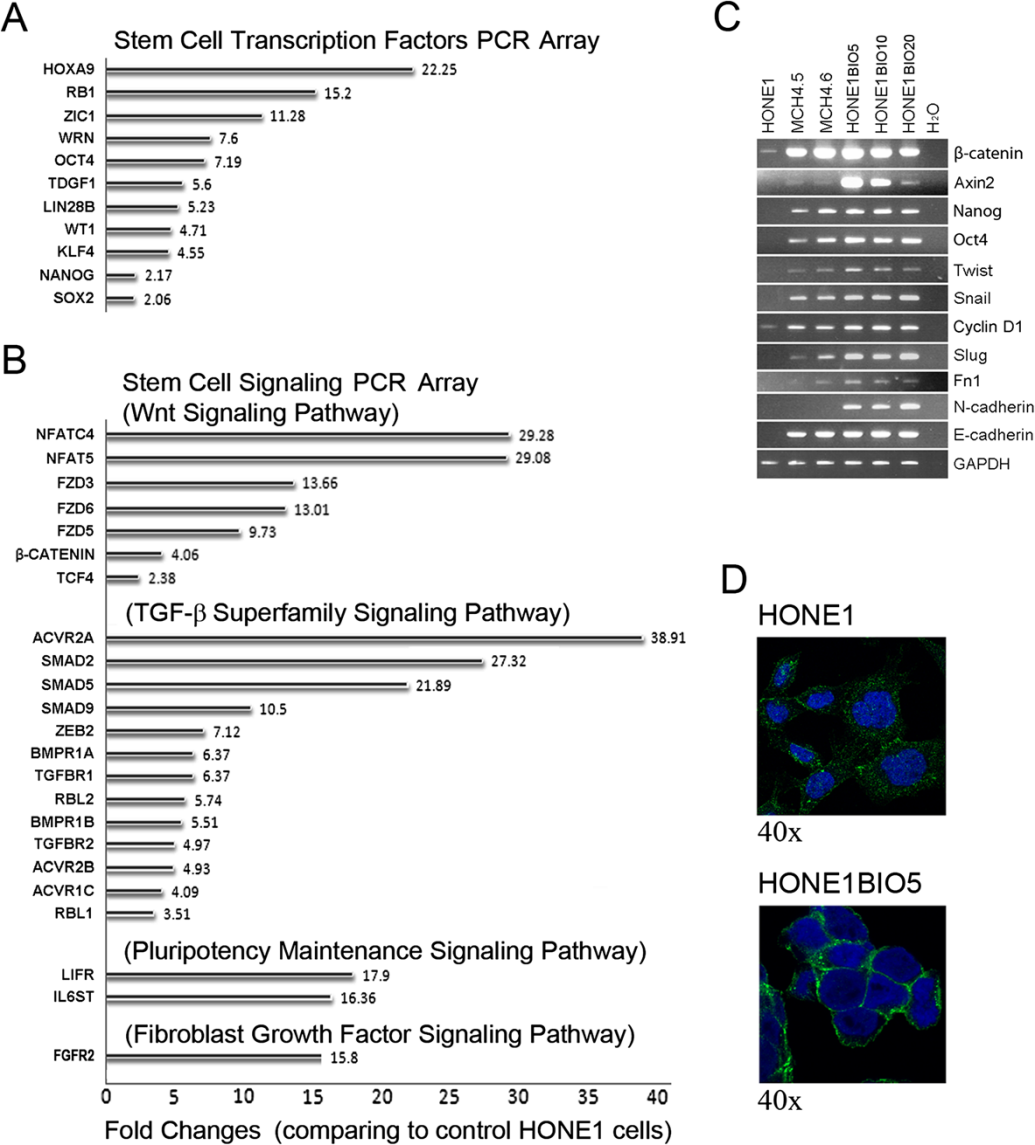
**Physiological β-catenin signaling triggers multiple signaling pathways in HONE1 hybrid cells, and BIO-induced Wnt signaling activities in HONE1 cells. A**. Stem Cell Transcription Factors Array shows major transcription factors that are up-regulated in HONE1 hybrid cells. **B**. The bottom panel demonstrates Stem Cell Signaling Array, showing that four signaling pathways are activated in HONE1 hybrid cells. **C**. RT-PCR analyses show that both β-catenin and *Axin2* are strongly expressed in BIO-treated cells. Except for *N-cadherin* that shows reduced levels in HONE1 hybrid cells (also see Figure 1C, expression for all tested genes, β-catenin*, Axin2, Nanog, Oct4, Twist, Snail, Cyclin D1, Slug, Fn1,* and *E-cadherin,* is up-regulated in both HONE1 hybrid (MCH4.5 and MCH4.6) and BIO-treated cells compared to mock-treated HONE1 cells. **D**. Immunofluorescence staining shows that expressed proteins in BIO-treated HONE1 cells (5 μM) are mainly accumulated in the membrane (40x magnification) as compared to parental HONE1 cells. This is also seen in HONE1 hybrid cells (Figures 1B and 1E).

The qPCR array results confirmed that pluripotency genes, *Sox2, Klf4, Oct4,* and *Nanog*, are clearly up-regulated in HONE1 hybrid cells, compared with parental HONE1 cells. In addition, many other relevant genes such as *HOXA9, RB1, ZIC1, WRN, TDGF1, LIN28B*, and *WT1* are considerably up-regulated in these HONE1 hybrid cells (Figure 4A), supporting the stem cell-like properties of these cells and activation of known genes related to self-renewal networks.

The exogenous copy of β-catenin in HONE1 hybrid cells induced signaling cascades involved in multiple pathways such as Wnt, TGF-β superfamily containing *BMP* and *Activin* receptors, pluripotency maintenance, and FGF signaling (Figure 4B). For Wnt signaling, enhanced expression of the non-canonical *NFAT* family and Wnt receptors, *FZD3, FZD5,* and *FZD6*, was detected in these cells. However, the Stem Cell Signaling Array did not detect significant changes of signals from Hedgehog and Notch pathways (Additional file 1: Table S1).

### BIO activation of Wnt signaling generates similar signaling activities as induced by chromosome 3 transfer in HONE1 cells

The GSK-3-specific inhibitor, Bromoindirubin-3’-oxime (BIO), activates Wnt signaling in stem cells and somatic cells [5,10,27,35] and this small molecule was, therefore, used to treat parental HONE1 cells to determine whether it had an effect on core stem gene signaling networks, similar to that seen in the HONE1 hybrid cells. As shown in Figure 4C, 5 μM BIO treatment induced the highest mRNA expression of β-catenin and *Axin2*. BIO treatment in HONE1 cells up-regulated mRNA expression of endogenous stem cell genes, *Nanog* and *Oct4*, and EMT markers and regulators such as *Twist, Snail, Cyclin D1*, *Slug*, *Fn1,* and *E-cadherin.* BIO treatment also caused an up-regulation of N-cadherin in HONE1 cells, suggesting that BIO treatment does not fully mimic chromosome 3 transfer effects of inducing physiological signaling in HONE1 cells. Bio-induced β-catenin accumulation was mainly detected at the membrane in treated cells (Figure 4D).

**Figure 5:**
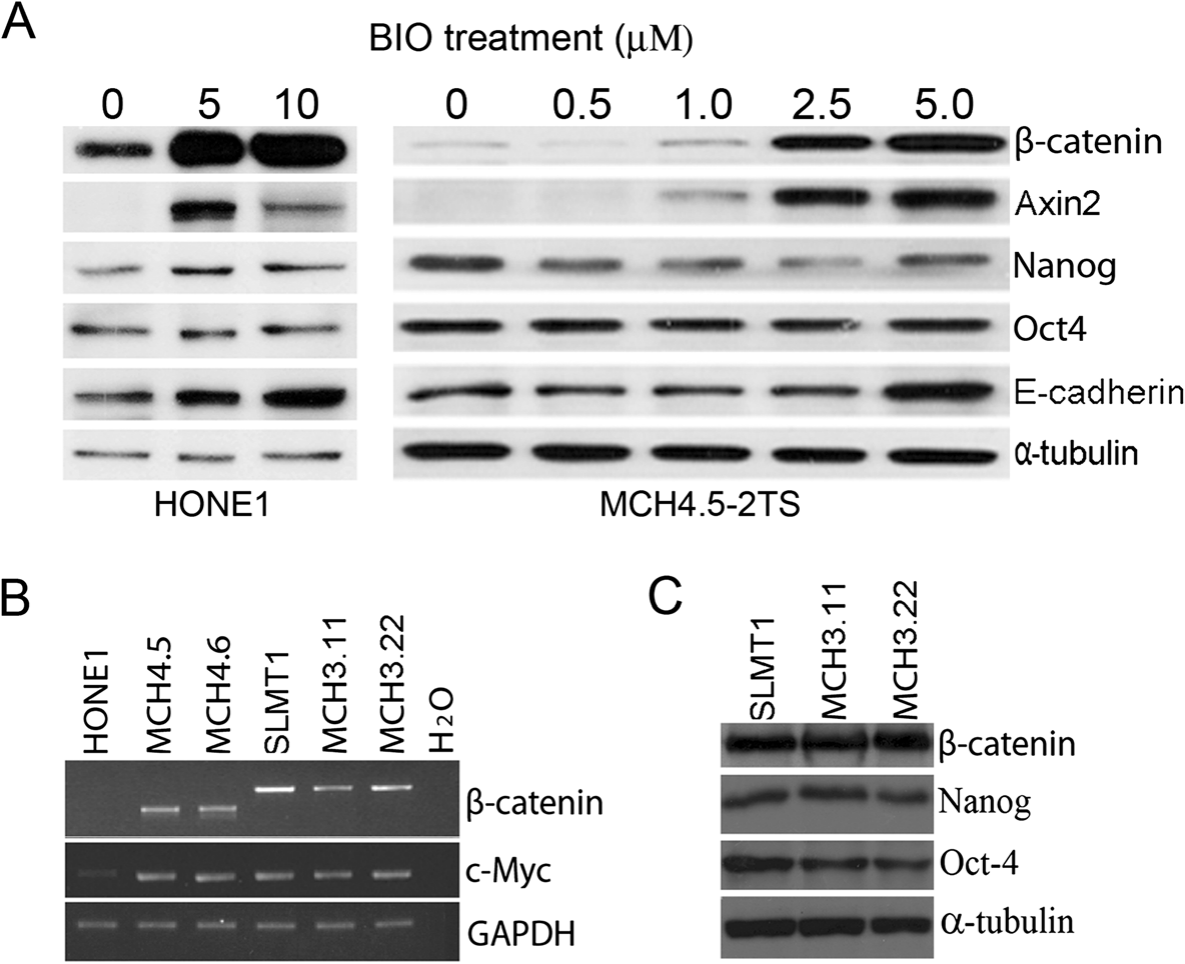
**The high levels of β-catenin signaling do not up-regulate Nanog and Oct4 expression in both HONE1 and SLMT1 cells. A**. Western blotting shows that both concentrations of 5 μM and 10 μM BIO treatment induce strong over-expression of β-catenin in treated cells, but 10 μM BIO treatment causes strong cytotoxic effects in cells and possible reduced expression of Axin2 in HONE1 cells. Compared to mock-treated HONE1 and MCH4.5-2TS cells, no obvious up-regulation of both Nanog and Oct4 expression is detected in β-catenin over-expressing cells, but E-cadherin is clearly up-regulated in both BIO-treated cell lines. **B**. SLMT1 cells have high levels of expression of β-catenin and do not respond to exogenous β-catenin signaling. RT-PCR analysis shows that HONE1 hybrid cells have an up-regulated expression of β-catenin and *c-Myc* compared to their parental HONE1 cells*,* but no up-regulated change of β-catenin (exons 3/5) and *c-Myc* is detected in SLMT1 and its hybrids, MCHs 3.11 and 3.22. **C**. Western blotting analyses do not detect changes of proteins β-catenin, Nanog, and Oct4 in SLMT1 hybrid cells, compared with their parental SLMT1 cells.

To verify the critical role that physiological signaling levels exert, BIO treatment was used to generate high, non-physiological levels of β-catenin protein expression in cells. Western blot analyses indicated that β-catenin, Axin2, and E-cadherin were strongly up-regulated in BIO-treated HONE1 cells. These results were also confirmed in MCH4.5-2TS cells that lost a transferred copy of *β-catenin* gene. Importantly, endogenous protein levels of Nanog and Oct4 were not up-regulated in β-catenin over-expressing HONE1 and MCH4.5-2TS cells (Figure 5A).

### Chromosome 3 transfer does not affect cells with high levels of endogenous β-catenin expression

To determine whether physiological β-catenin signaling might regulate other human cells, esophageal carcinoma SLMT1 cells were examined [36]. The endogenous levels of β-catenin expression in SLMT1 were higher than that of HONE1 cells, likely due to an alternative transcript of β-catenin gene in exons 3/5 in SLMT1 cell observed by RT-PCR and sequencing analyses (data not shown). The expressions of both β-catenin and c-Myc mRNAs remain unchanged in SLMT1 hybrid cells, MCH3.11/3.22 [36], with an introduced copy of chromosome 3, compared with their parental SLMT1 cells (Figure 5B). Nanog and Oct4 protein expression was also unaltered in SLMT1-derived hybrid cells (Figure 5C).

### Tumor suppressor gene-mediated pathways are activated in HONE1 hybrid cells

**Figure 6:**
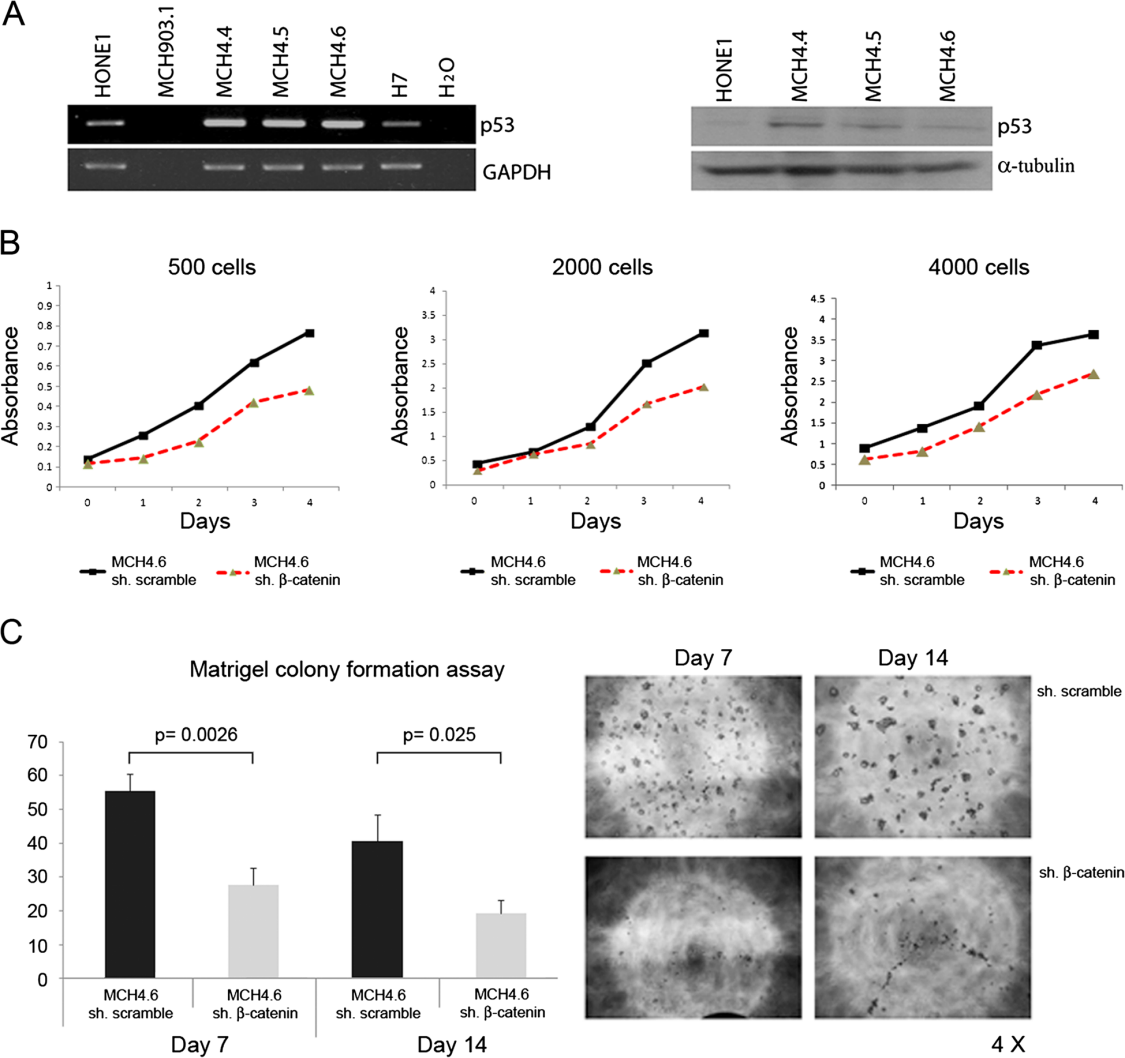
**The exogenous β-catenin signaling induces an up-regulation of *p53 *expression in HONE1 hybrid cells and influences proliferation of treated cells. A**. RT-PCR analysis shows that tumor suppressor *p53* is up-regulated by Wnt-signaling in HONE1 hybrid cells compared to parental HONE1 cells. Western blot analysis (right) confirms that *p53* expression is also up-regulated in HONE1 hybrid cells (MCH4.4/4.5/4.6). **B**. shRNA knockdown of β-catenin is associated with the reduction of tumor cell proliferation. MTT assays indicate cell proliferation rate is reduced in β-catenin shRNA infected HONE1 hybrid cells in different starting number of cells compared to scramble shRNA control cells. **C**. The colony formation assay was performed in matrigel. The colony formation ability in HONE1 hybrid cells with infected β-catenin shRNA is significantly inhibited on both days 7 and 14 compared to scramble shRNA control cells.

Because the tumor suppressor *p53* circuit communicates with the Wnt pathway and serves as a major target in stem cells [15,18], the expression of *p53* in HONE1 hybrid cells was examined at both the RNA and protein levels. Interestingly, *p53* was up-regulated and triggered by Wnt signaling in all these hybrid cells by both RT-PCR (Figure 6A) and Western blot analyses (Figure 6B). As described previously, other tumor suppressors, such as *RB1* and *WT1* (Figure 4A), were also activated in these cells, consistent with multiple tumor suppression pathways being activated following introduction of physiological Wnt/β-catenin signaling in HONE1 cells.

Since tumor suppression pathways were activated in HONE1 hybrid cells due to the up-regulation of Wnt/β-catenin signaling, it may affect growth ability of cells. To test these possibilities, MTT and colony formation assays in matrigel were performed with both stable scramble and β-catenin shRNA-infected HONE1 hybrid cells. The results indicated that tumor cell growth was inhibited by the knockdown of Wnt/β-catenin signaling in these cells (Figures 6B and 6C), reflecting that activation of tumor suppressive signaling has limited influence to control cell growth in HONE1 hybrid cells. However, Wnt/β-catenin signaling may stimulate cell growth during stemness transition process.

### Wnt signaling and stemness network activities are progressively up-regulated in sphere-forming cells

**Figure 7:**
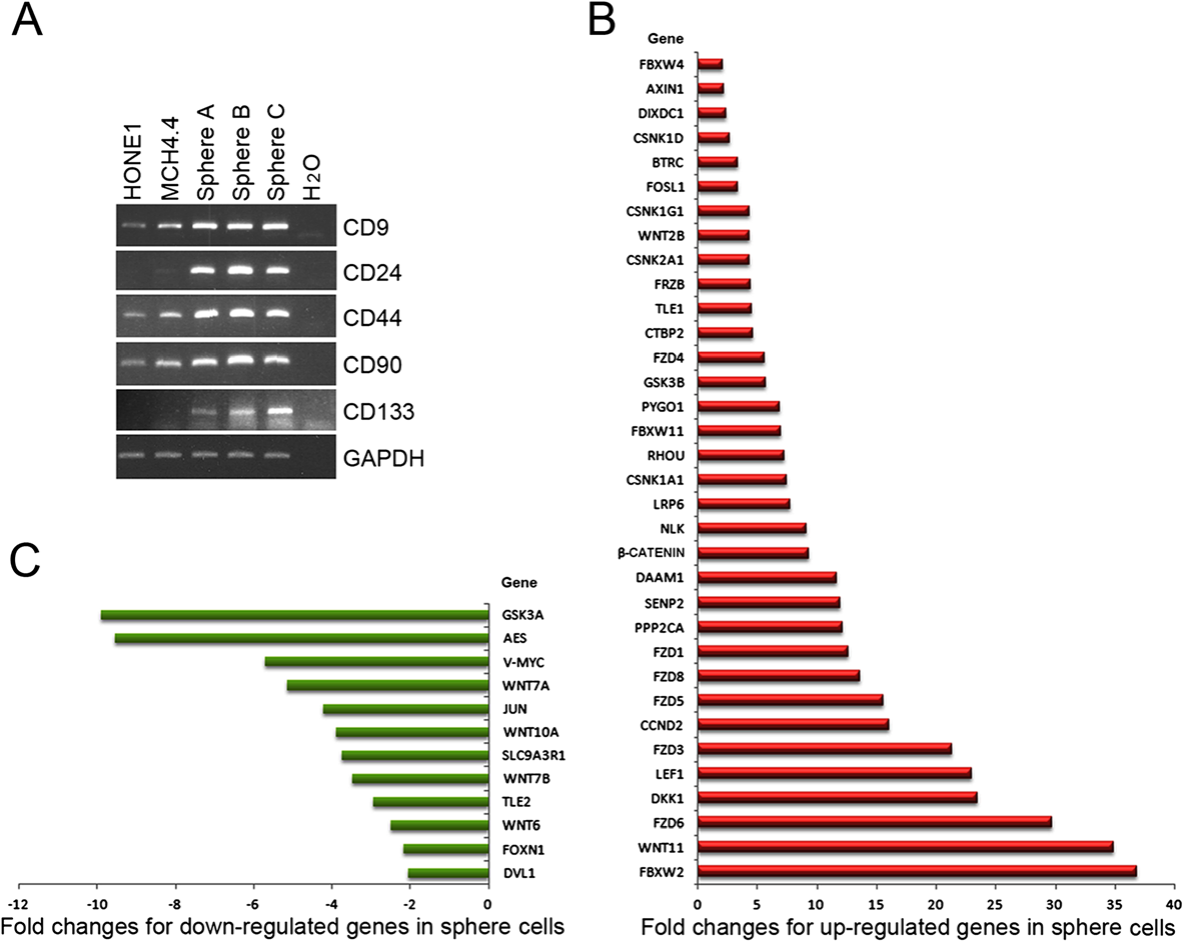
**Analyses of spheres derived from HONE1 hybrid cells. A**. RT-PCR analyses show that the expression of genes that encode CSC markers, CD9, CD24, CD44, CD90, and CD133, in sphere-forming cells (spheres **A**, **B**, and **C**) are progressively up-regulated compared to HONE1 and HONE1 hybrid cells. **B**. and **C**. Wnt Signaling Pathway qPCR Array assay lists the 34 up-regulated genes and 12 down-regulated genes in spheres, compared to HONE1 hybrid MCH4.4.

In the dedifferentiation progression of HONE1 to hybrid to sphere cells, RNA expression of four core stem cell genes, *Nanog*, *Oct4, Sox2,* and *Klf4*, was continuously up-regulated in spheres compared to derived hybrid cells (data not shown). Furthermore, some genes that encode CSC surface markers (Table S2) were either expressed or up-regulated by RT-PCR analysis in spheres, including CD9, CD24, CD44, CD90, and CD133. Notably, expression of genes that encode CD24 and CD133 was detected in sphere-forming cells derived from pooled large and small spheres from different experiments (Figure 7A).

Consistent with findings obtained from Stem Cell Signaling Array analysis (Figure 4A), many Wnt pathway components were activated, including both those in canonical and non-canonical signaling. In spheres, *LEF1* was further increased 23-fold, compared to hybrid cells. Other markedly up-regulated genes are *DKK1* (23.5-fold), *FZD6* (29.7-fold), *Wnt11* (34.9-fold)*,* and *FBXW2* (36.8-fold). Additionally, expression of some components of Wnt signaling, such as *GSK3A* (−9.9-fold) and the *TLE* repressor *AES* (−9.6-fold), were clearly inhibited in these spheres, as seen in Figures 7B and 7C.

## Discussion

Wnt signaling plays a substantial role in the control of development of several types of tissues through a dosage-dependent fashion. These regulated cells include crypt progenitor [28,37], hair follicle [38], and hematopoietic stem cells [39]. All these observations suggest that Wnt signaling is a dominant force in the control of proliferation of progenitor or stem cells. Our data further suggest that basic, moderate, physiological levels of Wnt signaling are sufficient for these cellular processes [3]. To investigate the hypothesized role of Wnt signaling in the regulation of stemness networks in human cells, HONE1, SLMT1, and their hybrid cell lines were used. As expected, strong activation of endogenous tumor suppressors *p53*, *RB1,* and *WT1* was detected in HONE1 hybrid cells, indicating that genomic integrity controlled by multiple tumor suppressor pathways within the parental HONE1 cells may be a key factor for maintaining a balanced environment to keep a low level of β-catenin expression [18-20].

Consistent with findings of both components of Wnt pathways and the core stem cell network being expressed or up-regulated in HONE1 hybrid cells, we found that the Wnt signal was clearly increased in HONE1 hybrid cells, as compared with untreated HONE1 cells. These *in vitro* observations were further confirmed *in vivo* with animal studies, indicating that there was consistent, up-regulated Wnt/β-catenin signaling in HONE1 hybrid cells.

To exclude the possibility that genes other than *β-catenin* on transferred chromosome 3 induced Wnt signaling in HONE1 hybrid cells, we specifically silenced β-catenin expression using β-catenin shRNA in these hybrid cells. Following the inhibition of β-catenin, expression of core stem cell genes and EMT markers was also decreased in the treated hybrid cells. These observations strongly indicate that β-catenin signaling, introduced by chromosome 3 transfer, is a dominant force in the regulation of both stemness and EMT networks in HONE1 cells, an expression pattern seen in other tissues [28,30,37-39].

Both chromosome 3 transfer and BIO treatment in HONE1 cells regulated EMT genes or its regulators. However, BIO only had similar, not identical biological effects as the chromosome 3 transfer, as evident in the control of cadherin switching. The Wnt signal controlling these EMT and cadherin networks was evidenced by the fact that β-catenin protein accumulated at the membranes, as seen in BIO-treated cells, hybrids with transferred chromosome 3, and recent stemness studies [40]. This may be another regulatory process for entry control of free β-catenin into the nucleus, but the detailed mechanism of nuclear localization of β-catenin and the critical role E-cadherin plays in these processes are still not fully understood [8,9,21,29,41,42].

The qPCR Array results confirmed that physiological β-catenin signaling triggered signals through additional pathways including pluripotency maintenance, FGF, and TGF-β superfamily signaling in HONE1 hybrid cells. For example, Smad2/5/9 and TGF-β receptors were clearly activated, which confirms previous reports that the TGF-β signaling pathway is associated with the pluripotency gene network and the EMT process [30,41]. Many embryonic development genes such as *Activin* receptor, *ACVR2A,* were activated following the introduction of physiological β-catenin signaling in treated cells. This suggests that there are additional signals such as *Activin* that develop during culture and may serve to drive the reprogramming or cell self-renewal processes induced by Wnt signaling. It is also notable that the LIFR*-* and IL6ST-mediated pluripotency maintenance pathways, as well as *BMP* receptors and *Smad* families, were activated in HONE1 hybrid cells. Since these signals have well-known links to self-renewal programs, their increased expression provides additional evidence that physiological Wnt/β-catenin signaling is involved in the regulation of the cell self-renewal networks [4,5,8,17,43].

The stemness transition of HONE1 cells, driven by physiological Wnt/β-catenin signaling, appears to be a progressive process leading to a de-differentiated state in spheres, as demonstrated by analysis of up-regulated expression of core stem cell genes. Furthermore, genes that encode surface markers, such as CD9, CD24, CD44, CD90, and CD133, in spheres were expressed or clearly up-regulated compared to hybrid cells. Many Wnt signaling components in sphere-forming cells were identified as being involved in this stemness transition process, compared to hybrid cells. More than two-fold up-regulation of expression was detected in 34 genes*,* which included canonical, non-canonical, and Wnt/Ca^+2^ pathways.

## Conclusions

These studies demonstrate that both dosage levels of β-catenin signaling and genetic background of tissues play critical roles in the regulation of self-renewal networks in human cells. There are very limited methods to explore physiological levels of Wnt/β-catenin signaling in current biology studies and the results of these studies indicate that haploid levels of expression of transferred β-catenin can induce signaling cascades that include multiple known pathways associated with reprogramming and self-renewal processes. Appropriate Wnt signaling may drive cancer cells into a progressive de-differentiated state, which generates expression profiles for exposure of the regulatory mechanism in stemness transition. These novel results clarify the relationships that are still controversial and rather unclear at present among Wnt signaling, stemness networks, EMT events, TSG pathways and expression of common cancer stem cell surface markers. These findings shed new light on a variety of cellular processes never before associated with physiological levels of β-catenin signaling that provide a framework for future studies to define the molecular mechanisms governing cell fate determination in human cells.

## Methods

### Cell lines and culture conditions

NPC HONE1 cells [44], mouse MCH903.1 cells containing human chromosome 3, three HONE1 hybrid cell lines (MCH4.4/4.5/4.6), and four TS cell lines (MCH4.5-1TS/4.5-2TS/4.6-1TS/4.6-2TS) were cultured as previously reported [11]. Esophageal carcinoma cell line, SLMT1 and its hybrid cells, MCH3.11/3.22, with transferred chromosome 3 were cultured as previously reported [36]. To minimize any possible change of gene expression influenced by culture conditions, environmental factors and genetic alterations in cultured cells [7], RNAs and proteins were obtained from early passages of hybrid cells and corresponding passages of parental cells for cell fusion. Human embryonic stem cells, H7, were obtained from the Stem Cell Core Facility of the University of Hong Kong and were maintained in Matrigel-covered plates and mTeSR1 culture medium, as suggested by the manufacturer (Stemcell Technologies, Vancouver, BC, Canada).

### BIO treatment

BIO (Stemgent, San Diego, USA) was stored and diluted following manufacturer’s instructions. Cells were grown in T25 flasks to 50% confluence and treated with BIO at concentrations of 0.5 μM, 1.0 μM, 2.5 μM, 5 μM, 10 μM, and 20 μM. Twenty-four hours later, both BIO-treated and mock-treated cells were washed in PBS and harvested for total RNA extraction.

### Sphere formation assay

HONE1, HONE1 hybrids, and HONE hybrids with infected β-catenin shRNA cells were plated in T25 flasks, coated with 0.1% gelatin at 10–30 viable cells/ml and grown in Dulbecco’s modified eagle medium (DMEM) supplemented with 10% FBS. Cells were allowed to grow until spheres appeared. Three independent experiments were performed. After spheres formed, non-spheroid cells surrounding the spheres were scratched off and washed away with PBS daily in order to maintain the sphere growth. Only 50% of the culture medium was changed each time. The number of spheres was recorded under microscopy from 45 to 90 days after seeding cells. The pooled large and small spheres were collected for further RNA analysis.

### RT^2^ Profiler PCR array analysis

Stem Cell Transcription Factors, Stem Cell Signaling, and Wnt Signaling Pathway PCR Arrays were obtained from SABIOSCIENCES (http://www.sabiosciences.com/Signal_Transduction.php), a Qiagen Company (Frederick, MD, USA). Multiple RNA expression (internal control and housekeeping genes) and genomic DNA contamination controls are included in these qPCR arrays. One μg of total RNA was used for the first strand cDNA synthesis reaction. All reaction procedures and data analyses were performed following the manufacturer’s manual (RT^2^ Profile PCR Array User Manual version 5.01) and provided analysis software (RT^2^ Profiler RCR Array Data Analysis version 3.5).

### Infection of shRNAs

Both shRNAs scramble [45] (Addgene plasmid 1864) and β-catenin [46] (Addgene plasmid 19761), were obtained from Addgene (Cambridge, MA USA). HEK-293T cells were incubated at 37°C overnight and transfected with shRNA plasmid, psPAX2 packaging plasmid, pMD2.G envelope plasmid, and FuGENE in DMEM medium, following Addgene’s protocol. Lentiviral particle solution from cultured cells was harvested 48 hours later. HONE1 hybrid cells were plated in T25 flasks overnight and grown to approximately 80-90% confluence. The following day, 1 ml infection media was added in 3 ml culture medium containing 8 μg/ml polybrene for 48 hours. The selective drug puromycin was added 24 hours later. Infected cells were expanded and the third to fifth passages were collected for subsequent RNA and protein analyses.

### Luciferase assay

HONE1 and HONE1 hybrid cells were transiently transfected using 1 μg of luciferase reporter plasmids (STOP) and control plasmids (SFOP) with the Lipofectamine 2000 (Invitrogen) transfection reagent according to manufacturer’s instructions. Cells were collected and lysed 48 hours post-transfection. The luciferase activity was assayed using the Luciferase Assay System (Promega). The β-galactosidase activity was assayed using Galacto-Light Plus System (Applied Biosystems, Life Technologies, Carlsbad, CA, USA). Both luciferase and β-galactosidase activities were measured using a TD20/20 luminometer (Turner Biosystems). STOP/SFOP ratio of luciferase reporter activities was normalized using β-galactosidase activities [47,48].

### RT-PCR and qPCR analyses

Total RNA was extracted using TRIzol reagent (Invitrogen, Carlsbad, CA, USA). One microgram of total RNA was reverse-transcribed with M-MLV Reverse Transcriptase (USB, Cleveland, OH, USA), as previously described [48]. All RNAs were treated with DNAse. The primers and RT-PCR and qPCR conditions are summarized in Additional file 1: Table S2. Human *GAPDH* was used as an internal control for all RT-PCR reactions. For qPCR analysis, triplicate PCR reactions were performed using the LightCycler 480 Real-Time PCR Instrument (Roche Diagnositics GmbH, Mannheim, Germany).

### Flow cytometry

Antibodies against CD24 and CD44 (BD Pharmingen) were used for FACS analyses. To sort HONE1 and HONE1 hybrid cells, the collected cells were resuspended in Pharmingen Stain Buffer and stained with antibodies for 30 min. The antibody-positive cells were sorted by the BD FACSAria Cell Sorter (San Jose, CA, USA), according to the protocols provided by the manufacturer.

### Cell proliferation assay

The cell proliferation ability was studied using the 3-(4,5-dimethylthiazol-2-yl)-2,5-diphenyl-tetrazolium bromide (MTT) assay. Briefly, 0.5 × 10^3^, 2.0 × 10^3^, and 4.0 × 10^3^ cells were seeded in 96-well plate. The cell proliferation rate was measured consecutively for 5 days. Thirty μl of 5 mg/ml filtered MTT solution (Sigma Chemical Co., St. Louis, MO) was added into the cells and incubated at 37°C for 4 hours. The absorbance at 570 nm was recorded with Multiskan FC Microplate Photometer (Thermo Scientific Inc., MA, USA).

### Three-dimensional matrigel colony formation assays

A total of 1,000 cells was seeded on the top of Matrigel (BD Biosciences) and cultured for 14 days in 96-well plate. Images were captured at 4x and 10x magnification using Nikon Eclipse Ti inverted microscope (Nikon Instruments Inc., NY, USA) and analyzed with SPOT Advanced software (Diagnostic Instruments Inc., MI, USA). Colonies in 3 fields at 10x magnifications were counted and reported as the average number of colonies on the day 7 and 14.

### Western blotting and immunofluorescence staining

Cells were seeded onto a 150 mm culture plate and were then scraped from the plate for Western blotting, which were performed as previously described [49]. The primary antibodies for Western blot analyses are summarized in Additional file 1: Table S3. Cells for immunofluorescence staining of β-catenin were grown on glass coverslips and incubated with primary and secondary antibodies for overnight and one hour, respectively, and analyzed by confocal microscopy (LSM 710, Zeiss, Germany).

### Animal assay and immunohistochemical staining

Both HONE1 and HONE1 hybrid cells were injected into nude mice as previously described [11,12]. Tumors derived from both HONE1 and HONE1 hybrid cells were excised for both tissue culture in selective medium and paraffin-embedded for immunohistochemistry analysis using antibodies (β-catenin, E-cadherin, and Cyclin D1) (Additional file 1: Table S3), as described previously [48]. All animal experiments were approved by the Government of Hong Kong Special Administrative Region and University of Hong Kong.

## Abbreviations

BIO: Bromoindirubin-3’-oxime; CSC: Cancer stem cells; DMEM: Dulbecco’s modified eagle medium; EMT: Epithelial-mesenchymal transition; FGF: Fibroblast growth factor; MHC: Microcell hybrid cell; MTT: 3-(4,5-dimethylthiazol-2-yl)-2,5-diphenyl-tetrazolium bromide; NPC: Nasopharyngeal carcinoma; ShRNA: Short hairpin RNA; TGF-β: Transforming growth factor-β; TS: Tumor segregants; TSG: Tumor suppressor gene.

## Competing interest

The authors declare that they have no competing interest.

## Authors’ contributions

YC designed research. YC performed molecular, cell culture and animal studies. AKLC, JMYK and YPP carried out Western and immunofluorescence staining. YPP and YC performed Luciferase, qPCR array and *in vitro* growth assays. PMC performed immunohistochemical staining. JMYK and PHYL performed studies in SLMT1 and SLMT1 hybrid cells. YC, MLL and MLW contributed cell lines and reagents. YC, AKLC, JMYK, YPP, MLL and MLW analyzed data. YC, MLL and MLW wrote manuscript. All authors have read and approved the final manuscript.

## Supplementary Material

Additional file 1 **Table S1.** Expression changes in HONE1 hybrid Cells (compared to parental HONE1 cells) assayed by Stem Cell Signaling PCR Array. **Table S2.** Primers used in RT-PCR and qPCR analyses.** Table S3.** Antibodies used. Click here for file

## References

[B1] Fodde R Brabletz T Wnt/beta-catenin signaling in cancer stemness and malignant behavior Curr Opin Cell Biol 2007 19 2 150 158 10.1016/j.ceb.2007.02.007 17306971

[B2] Kikuchi A Yamamoto H Sato A Selective activation mechanisms of Wnt signaling pathways Trends Cell Biol 2009 19 3 119 129 10.1016/j.tcb.2009.01.003 19208479

[B3] Reya T Clevers H Wnt signalling in stem cells and cancer Nature 2005 434 7035 843 850 10.1038/nature03319 15829953

[B4] ten Berge D Kurek D Blauwkamp T Koole W Maas A Eroglu E Siu RK Nusse R Embryonic stem cells require Wnt proteins to prevent differentiation to epiblast stem cells Nat Cell Biol 2011 13 9 1070 1075 10.1038/ncb2314 21841791 PMC4157727

[B5] Dravid G Ye Z Hammond H Chen G Pyle A Donovan P Yu X Cheng L Defining the role of Wnt/beta-catenin signaling in the survival, proliferation, and self-renewal of human embryonic stem cells Stem Cells 2005 23 10 1489 1501 10.1634/stemcells.2005-0034 16002782

[B6] Kielman MF Rindapaa M Gaspar C van Poppel N Breukel C van Leeuwen S Taketo MM Roberts S Smits R Fodde R Apc modulates embryonic stem-cell differentiation by controlling the dosage of beta-catenin signaling Nat Genet 2002 32 4 594 605 10.1038/ng1045 12426568

[B7] Vermeulen L De Sousa EMF van der Heijden M Cameron K de Jong JH Borovski T Tuynman JB Todaro M Merz C Rodermond H Wnt activity defines colon cancer stem cells and is regulated by the microenvironment Nat Cell Biol 2010 12 5 468 476 10.1038/ncb2048 20418870

[B8] Clevers H Nusse R Wnt/beta-catenin signaling and disease Cell 2012 149 6 1192 1205 10.1016/j.cell.2012.05.012 22682243

[B9] Wray J Hartmann C WNTing embryonic stem cells Trends Cell Biol 2012 22 3 159 168 10.1016/j.tcb.2011.11.004 22196214

[B10] Davidson KC Adams AM Goodson JM McDonald CE Potter JC Berndt JD Biechele TL Taylor RJ Moon RT Wnt/beta-catenin signaling promotes differentiation, not self-renewal, of human embryonic stem cells and is repressed by Oct4 Proc Natl Acad Sci USA 2012 109 12 4485 4490 10.1073/pnas.1118777109 22392999 PMC3311359

[B11] Cheng Y Poulos NE Lung ML Hampton G Ou B Lerman MI Stanbridge EJ Functional evidence for a nasopharyngeal carcinoma tumor suppressor gene that maps at chromosome 3p21.3 Proc Natl Acad Sci USA 1998 95 6 3042 3047 10.1073/pnas.95.6.3042 9501212 PMC19691

[B12] Cheng Y Stanbridge EJ Kong H Bengtsson U Lerman MI Lung ML A functional investigation of tumor suppressor gene activities in a nasopharyngeal carcinoma cell line HONE1 using a monochromosome transfer approach Genes Chromosomes Cancer 2000 28 1 82 91 10.1002/(SICI)1098-2264(200005)28:1<82::AID-GCC10>3.0.CO;2-8 10738306

[B13] Sun Y Hegamyer G Cheng YJ Hildesheim A Chen JY Chen IH Cao Y Yao KT Colburn NH An infrequent point mutation of the p53 gene in human nasopharyngeal carcinoma Proc Natl Acad Sci USA 1992 89 14 6516 6520 10.1073/pnas.89.14.6516 1631151 PMC49532

[B14] Sun Y Hegamyer G Colburn NH Nasopharyngeal carcinoma shows no detectable retinoblastoma susceptibility gene alterations Oncogene 1993 8 3 791 795 8437863

[B15] Hong H Takahashi K Ichisaka T Aoi T Kanagawa O Nakagawa M Okita K Yamanaka S Suppression of induced pluripotent stem cell generation by the p53-p21 pathway Nature 2009 460 7259 1132 1135 10.1038/nature08235 19668191 PMC2917235

[B16] Pajcini KV Corbel SY Sage J Pomerantz JH Blau HM Transient inactivation of Rb and ARF yields regenerative cells from postmitotic mammalian muscle Cell Stem Cell 2010 7 2 198 213 10.1016/j.stem.2010.05.022 20682446 PMC2919350

[B17] He S Nakada D Morrison SJ Mechanisms of stem cell self-renewal Annu Rev Cell Dev Biol 2009 25 377 406 10.1146/annurev.cellbio.042308.113248 19575646

[B18] Damalas A Ben-Ze'ev A Simcha I Shtutman M Leal JF Zhurinsky J Geiger B Oren M Excess beta-catenin promotes accumulation of transcriptionally active p53 EMBO J 1999 18 11 3054 3063 10.1093/emboj/18.11.3054 10357817 PMC1171387

[B19] Kim NH Kim HS Kim NG Lee I Choi HS Li XY Kang SE Cha SY Ryu JK Na JM p53 and microRNA-34 are suppressors of canonical Wnt signaling Sci Signal 2011 4 197 ra71 10.1126/scisignal.2001744 22045851 PMC3447368

[B20] Lee KH Li M Michalowski AM Zhang X Liao H Chen L Xu Y Wu X Huang J A genomewide study identifies the Wnt signaling pathway as a major target of p53 in murine embryonic stem cells Proc Natl Acad Sci USA 2010 107 1 69 74 10.1073/pnas.0909734107 20018659 PMC2806696

[B21] Lyashenko N Winter M Migliorini D Biechele T Moon RT Hartmann C Differential requirement for the dual functions of beta-catenin in embryonic stem cell self-renewal and germ layer formation Nat Cell Biol 2011 13 7 753 761 10.1038/ncb2260 21685890 PMC3130149

[B22] Sokol SY Maintaining embryonic stem cell pluripotency with Wnt signaling Development 2011 138 20 4341 4350 10.1242/dev.066209 21903672 PMC3177306

[B23] Wray J Kalkan T Gomez-Lopez S Eckardt D Cook A Kemler R Smith A Inhibition of glycogen synthase kinase-3 alleviates Tcf3 repression of the pluripotency network and increases embryonic stem cell resistance to differentiation Nat Cell Biol 2011 13 7 838 845 10.1038/ncb2267 21685889 PMC3160487

[B24] Yi F Pereira L Hoffman JA Shy BR Yuen CM Liu DR Merrill BJ Opposing effects of Tcf3 and Tcf1 control Wnt stimulation of embryonic stem cell self-renewal Nat Cell Biol 2011 13 7 762 770 10.1038/ncb2283 21685894 PMC3129424

[B25] Merrill BJ Develop-WNTs in somatic cell reprogramming Cell Stem Cell 2008 3 5 465 466 10.1016/j.stem.2008.10.011 18983957

[B26] Katoh M WNT signaling pathway and stem cell signaling network Clin Cancer Res 2007 13 14 4042 4045 10.1158/1078-0432.CCR-06-2316 17634527

[B27] Lluis F Pedone E Pepe S Cosma MP Periodic activation of Wnt/beta-catenin signaling enhances somatic cell reprogramming mediated by cell fusion Cell Stem Cell 2008 3 5 493 507 10.1016/j.stem.2008.08.017 18983965

[B28] van de Wetering M Sancho E Verweij C de Lau W Oving I Hurlstone A van der Horn K Batlle E Coudreuse D Haramis AP The beta-catenin/TCF-4 complex imposes a crypt progenitor phenotype on colorectal cancer cells Cell 2002 111 2 241 250 10.1016/S0092-8674(02)01014-0 12408868

[B29] Chen T Yuan D Wei B Jiang J Kang J Ling K Gu Y Li J Xiao L Pei G E-cadherin-mediated cell-cell contact is critical for induced pluripotent stem cell generation Stem Cells 2010 28 8 1315 1325 10.1002/stem.456 20521328

[B30] Scheel C Eaton EN Li SH Chaffer CL Reinhardt F Kah KJ Bell G Guo W Rubin J Richardson AL Paracrine and autocrine signals induce and maintain mesenchymal and stem cell states in the breast Cell 2011 145 6 926 940 10.1016/j.cell.2011.04.029 21663795 PMC3930331

[B31] Thomson JA Itskovitz-Eldor J Shapiro SS Waknitz MA Swiergiel JJ Marshall VS Jones JM Embryonic stem cell lines derived from human blastocysts Science 1998 282 5391 1145 1147 9804556 10.1126/science.282.5391.1145

[B32] Cheng Y Chakrabarti R Garcia-Barcelo M Ha TJ Srivatsan ES Stanbridge EJ Lung ML Mapping of nasopharyngeal carcinoma tumor-suppressive activity to a 1.8-megabase region of chromosome band 11q13 Genes Chromosomes Cancer 2002 34 1 97 103 10.1002/gcc.10048 11921287

[B33] Lung HL Cheung AK Ko JM Cheng Y Stanbridge EJ Lung ML Deciphering the molecular genetic basis of NPC through functional approaches Semin Cancer Biol 2012 22 2 87 95 10.1016/j.semcancer.2011.11.002 22154888

[B34] Cheng Y Ko JM Lung HL Lo PH Stanbridge EJ Lung ML Monochromosome transfer provides functional evidence for growth-suppressive genes on chromosome 14 in nasopharyngeal carcinoma Genes Chromosomes Cancer 2003 37 4 359 368 10.1002/gcc.10228 12800147

[B35] Sato N Meijer L Skaltsounis L Greengard P Brivanlou AH Maintenance of pluripotency in human and mouse embryonic stem cells through activation of Wnt signaling by a pharmacological GSK-3-specific inhibitor Nat Med 2004 10 1 55 63 10.1038/nm979 14702635

[B36] Lo PH Leung AC Kwok CY Cheung WS Ko JM Yang LC Law S Wang LD Li J Stanbridge EJ Identification of a tumor suppressive critical region mapping to 3p14.2 in esophageal squamous cell carcinoma and studies of a candidate tumor suppressor gene, ADAMTS9 Oncogene 2007 26 1 148 157 10.1038/sj.onc.1209767 16799631

[B37] Batlle E Henderson JT Beghtel H van den Born MM Sancho E Huls G Meeldijk J Robertson J van de Wetering M Pawson T Beta-catenin and TCF mediate cell positioning in the intestinal epithelium by controlling the expression of EphB/ephrinB Cell 2002 111 2 251 263 10.1016/S0092-8674(02)01015-2 12408869

[B38] Lowry WE Blanpain C Nowak JA Guasch G Lewis L Fuchs E Defining the impact of beta-catenin/Tcf transactivation on epithelial stem cells Genes Dev 2005 19 13 1596 1611 10.1101/gad.1324905 15961525 PMC1172065

[B39] Luis TC Naber BA Roozen PP Brugman MH de Haas EF Ghazvini M Fibbe WE van Dongen JJ Fodde R Staal FJ Canonical wnt signaling regulates hematopoiesis in a dosage-dependent fashion Cell Stem Cell 2011 9 4 345 356 10.1016/j.stem.2011.07.017 21982234

[B40] Ocana OH Corcoles R Fabra A Moreno-Bueno G Acloque H Vega S Barrallo-Gimeno A Cano A Nieto MA Metastatic colonization requires the repression of the epithelial-mesenchymal transition inducer prrx1 Cancer Cell 2012 22 6 709 724 10.1016/j.ccr.2012.10.012 23201163

[B41] Li R Liang J Ni S Zhou T Qing X Li H He W Chen J Li F Zhuang Q A mesenchymal-to-epithelial transition initiates and is required for the nuclear reprogramming of mouse fibroblasts Cell Stem Cell 2010 7 1 51 63 10.1016/j.stem.2010.04.014 20621050

[B42] Soncin F Mohamet L Eckardt D Ritson S Eastham AM Bobola N Russell A Davies S Kemler R Merry CL Abrogation of E-cadherin-mediated cell-cell contact in mouse embryonic stem cells results in reversible LIF-independent self-renewal Stem Cells 2009 27 9 2069 2080 10.1002/stem.134 19544408

[B43] Cheung AKL Phoon YP Lung HL Ko JMY Cheng Y Lung ML Cheng Y Roles of tumor suppressor signaling on reprogramming and stemness transition in somatic cells Future aspects of tumor suppressor gene 2013 Croatia: InTech 75 96

[B44] Glaser R Zhang HY Yao KT Zhu HC Wang FX Li GY Wen DS Li YP Two epithelial tumor cell lines (HNE-1 and HONE-1) latently infected with Epstein-Barr virus that were derived from nasopharyngeal carcinomas Proc Natl Acad Sci USA 1989 86 23 9524 9528 10.1073/pnas.86.23.9524 2556716 PMC298529

[B45] Sarbassov DD Guertin DA Ali SM Sabatini DM Phosphorylation and regulation of Akt/PKB by the rictor-mTOR complex Science 2005 307 5712 1098 1101 10.1126/science.1106148 15718470

[B46] Firestein R Bass AJ Kim SY Dunn IF Silver SJ Guney I Freed E Ligon AH Vena N Ogino S CDK8 is a colorectal cancer oncogene that regulates beta-catenin activity Nature 2008 455 7212 547 551 10.1038/nature07179 18794900 PMC2587138

[B47] Atcha FA Syed A Wu B Hoverter NP Yokoyama NN Ting JH Munguia JE Mangalam HJ Marsh JL Waterman ML A unique DNA binding domain converts T-cell factors into strong Wnt effectors Mol Cell Biol 2007 27 23 8352 8363 10.1128/MCB.02132-06 17893322 PMC2169181

[B48] Arce L Yokoyama NN Waterman ML Diversity of LEF/TCF action in development and disease Oncogene 2006 25 57 7492 7504 10.1038/sj.onc.1210056 17143293

[B49] Cheung AK Lung HL Ko JM Cheng Y Stanbridge EJ Zabarovsky ER Nicholls JM Chua D Tsao SW Guan XY Chromosome 14 transfer and functional studies identify a candidate tumor suppressor gene, mirror image polydactyly 1, in nasopharyngeal carcinoma Proc Natl Acad Sci USA 2009 106 34 14478 14483 10.1073/pnas.0900198106 19667180 PMC2732794

