# Supplementary material for: Physiological β-catenin signaling controls self-renewal networks and generation of stem-like cells from nasopharyngeal carcinoma
Source: BMC Cell Biol. 2013 Sep 27;14:44. doi: 10.1186/1471-2121-14-44 (PMC3819748; doi:10.1186/1471-2121-14-44)
Supplement: Additional file 1 — Table S1. Expression changes in HONE1 hybrid Cells (compared to parental HONE1 cells) assayed by Stem Cell Signaling PCR Array. Table S2. Primers used in RT-PCR and qPCR analyses. Table S3. Antibodies used. [file 1471-2121-14-44-S1.pdf]

Additional material

Additional file 1: Table S1. Expression changes in HONE1 hybrid Cells (compared to parental HONE1 cells) assayed by Stem Cell Signaling PCR Array

Gene Symbol	Fold change	Pathways
ACVR1	3.6351	TGF-β Superfamily Signaling
ACVR1B	1.0512	TGF-β Superfamily Signaling
ACVR1C	4.0897	TGF-β Superfamily Signaling
ACVR2A	38.9081	TGF-β Superfamily Signaling
ACVR2B	4.9314	TGF-β Superfamily Signaling
ACVRL1	0.8134	TGF-β Superfamily Signaling
AMHR2	1.8302	TGF-β Superfamily Signaling
BCL9	0.8901	Wnt Signaling
BCL9L	1.948	Wnt Signaling
BMPR1A	6.3731	TGF-β Superfamily Signaling
BMPR1B	5.5098	TGF-β Superfamily Signaling
BMPR2	2.0591	TGF-β Superfamily Signaling
CREBBP	3.9231	TGF-β Superfamily Signaling
CTNNB1 (β-CATENIN)	4.0615	Wnt Signaling
E2F5	1.6495	TGF-β Superfamily Signaling
ENG	0.2817	TGF-β Superfamily Signaling
EP300	1.7802	TGF-β Superfamily Signaling
FGFR1	1.0439	Fibroblast Growth Factor (FGF) Signaling
FGFR2	15.8016	Fibroblast Growth Factor (FGF) Signaling
FGFR3	0.7032	Fibroblast Growth Factor (FGF) Signaling
FGFR4	0.1951	Fibroblast Growth Factor (FGF) Signaling
FZD1	1.5933	Wnt Signaling
FZD2	0.2256	Wnt Signaling
FZD3	13.6611	Wnt Signaling
FZD4	2.0734	Wnt Signaling
FZD5	9.727	Wnt Signaling
FZD6	13.0141	Wnt Signaling
FZD7	0.4836	Wnt Signaling
FZD8	3.3683	Wnt Signaling
FZD9	0.5996	Wnt Signaling
GLI1	0.5556	Hedgehog Signaling
GLI2	0.4803	Hedgehog Signaling
GLI3	1.6725	Hedgehog Signaling
IL6ST	16.3589	Pluripotency Maintenance

LEF1	1.6609	Wnt Signaling
LIFR	17.9014	Pluripotency Maintenance
LRP5	0.8247	Wnt Signaling
LRP6	3.4629	Wnt Signaling
LTBP1	3.9779	TGF-β Superfamily Signaling
LTBP2	2.8919	TGF-β Superfamily Signaling
LTBP3	0.5042	TGF-β Superfamily Signaling
LTBP4	2.6982	TGF-β Superfamily Signaling
NCSTN	0.9474	Notch Signaling
NFAT5	29.0809	Wnt Signaling
NFATC1	0.6338	Wnt Signaling
NFATC2	0.5404	Wnt Signaling
NFATC3	1.9616	Wnt Signaling
NFATC4	29.2832	Wnt Signaling
NOTCH1	0.3615	Notch Signaling
NOTCH2	1.3398	Notch Signaling
NOTCH3	1.7195	Notch Signaling
NOTCH4	1.5713	Notch Signaling
PSEN1	1.0014	Notch Signaling
PSEN2	0.5832	Notch Signaling
PSENEN	0.3516	Notch Signaling
PTCH1	1.2243	Hedgehog Signaling
PTCHD2	1.4661	Hedgehog Signaling
PYGO2	0.1499	Wnt Signaling
RBL1	3.5113	TGF-β Superfamily Signaling
RBL2	5.7438	TGF-β Superfamily Signaling
RBPJL	1.2852	Notch Signaling
RGMA	0.4938	TGF-β Superfamily Signaling
SMAD1	2.912	TGF-β Superfamily Signaling
SMAD2	27.3222	TGF-β Superfamily Signaling
SMAD3	2.7359	TGF-β Superfamily Signaling
SMAD4	2.5527	TGF-β Superfamily Signaling
SMAD5	21.887	TGF-β Superfamily Signaling
SMAD6	0.274	TGF-β Superfamily Signaling
SMAD7	1.7802	TGF-β Superfamily Signaling
SMAD9	10.4977	TGF-β Superfamily Signaling
SMO	1.5823	Hedgehog Signaling
SP1	1.1111	TGF-β Superfamily Signaling
STAT3	1.3775	Pluripotency Maintenance
SUFU	0.6122	Hedgehog Signaling
TCF7 (TCF1)	1.2941	Wnt Signaling

TCF7L1 (TCF3)	1.7195	Wnt Signaling
TCF7L2 (TCF4)	2.3817	Wnt Signaling
TGFBR1	6.3731	TGF-β Superfamily Signaling
TGFBR2	4.9657	TGF-β Superfamily Signaling
TGFBR3	3.7633	TGF-β Superfamily Signaling
TGFBRAP1	0.8022	TGF-β Superfamily Signaling
VANGL2	1.1991	Wnt Signaling
ZEB2	7.1206	TGF-β Superfamily Signaling

http://sabiosciences.com/rt_pcr_product/HTML/PAHS-047A.html

Additional file 2: Table S2. Primers used in RT-PCR and qPCR analyses

Name	Sequences	Product size (bp)	Annealing temperature (℃)	Reference[d]
GAPDH F[a]	GAAGGTGAAGGTCGGAGTC	226	55	
GAPDH R[a]	GAAGATGGTGATGGGATTTC			
Sox2 F	CAGTACAACTCCATGACCAGCTCG	277	55	
Sox2 R	AGTGCTGGGACATGTGAAGTCTGC			
qSOX2 F[a]	TGCTGCCTCTTTAAGACTAGGAC	75	60	
qSOX2 R[a]	CCTGGGGCTCAAACTTCTCT			
CTNNB1 F	TAGAGGCTCTTGTGCGTACTGTCC	385	55	
CTNNB1 R	CCTTCAACTATTTCTTCCATGCGG			
CTNNB1 1F[b]	GTCCTTCACTCAAGAACAAGTAGC	474	55	
CTNNB1 1R[b]	GCCAGTAAGCCCTCACGATGATGG			
qCTNNB1 F[a]	GGCTTGGAATGAGACTGCTGAT	115	60	
qCTNNB1 R[a]	CTGGCCATATCCACCAGAGTG			
Klf4 F	CGAACCCACACAGGTGAGAA	68	55	[1]
Klf4 R	GAGCGGGCGAATTTCCAT			
qKLF4 F[a]	GGGAGAAGACACTGCGTCA	88	62	
qKLF4 R[a]	GGAAGCACTGGGGGAAGT			
c-Myc F	AGGCCACAGCAAACCTCCTC	300	59	
c-Myc R	TCCAACTCCGGGATCTGGTC			
Axin2 F	CGCAGCAGTTTGGCGGCAGCA	350	55	
Axin2 R	AGGGTCCTGGGTGAACAGGTGGG			
Tcf1 F	CAGTGTCCTCCAGCAGCCTG	191	60	
Tcf1 R	GCTGCTGCCCTCATCACCCC			
Tcf3 F	GAAATCACCAGTCACCGTGAAA	251	53	[2]
Tcf3 R	ACCAGGTTGGGTAGAGCTGCG			
Nanog F	ACTGGCCGAAGAATAGCAATGG	331	55	
Nanog R	AAGGCAGCCTCCAAGTCACTGG			
UPL-NANOG-F1[a]	ATGCCTCACACGGAGACTGT	66	60	
UPL-NANOG-R1[a]	AGGGCTGTCCTGAATAAGCA			
OCT4 F	CTTGCTGCAGAAGTGGGTGGAGGAA	169	55	NIH website[c]
OCT4 R	CTGCAGTGTGGGTTTCGGGCA			

UPL-OCT4-F1 [a]	CCTGTCTCCGTCACCACTCT	118	60	
UPL-OCT4-R1 [a]	GGCACAAACTCCAGGTTTTC			
p53 F	TACTGGGACGGAACAGCTTTGAGG	276	55	
p53 R	CAGCCTGGGCATCCTTGAGTTCC			
CD9 F	GTGCATGCTGGGACTGTTCTTCGGCTTC	220	55	[3]
CD9 R	CACGCCCCCAGCCAAACCACAGCAG			
CD24 F	TGAAGAACATGTGAGAGGTTTGAC	208	55	[4]
CD24 R	GAAAACTGAATCTCCATTCCACAA			
CD44s5	GCAGCACTTCAGGAGGTTACAT	272	55	[5]
CD44s11	CAAGAGGGATGCCAAGATGAT			
Thy1 F (CD90)	GACCCGTGAGACAAAGAAGC	235	50	[6]
Thy1 R (CD90)	GCCCTCACACTTGACCAGTT			
PROM1 F (CD133)	AATGCACCAGCGACAGAAG	194	55	[7]
PROM1 R (CD133)	CATTCAAGAGAGTTCGCAAGTC			
Twist1 F	GGAGTCCGCAGTCTTACGAG	201	55	[8]
Twist1 R	TCTGGAGGACCTGGTAGAGG			
Snail1 F	CTGCTACAAGGCCATGTCCGGACC	206	55	
Snail1 R	CATCCTGAGCAGCCGGACTCTTGG			
CyclinD1 F	CTGTGCTGCGAAGTGGAAACCAT	233	55	
CyclinD1 R	TTCATGGCCAGCGGGAAGACCTC			
hSlug-5	GGGGAGAAGCCTTTTTCTTG	158	55	[9]
hSlug-3	TCCTCATGTTTGTGCAGGAG			
hFn1-5	CAGTGGGAGACCTCGAGAAG	168	53	[9]
hFn1-3	TCCCTCGGAACATCAGAAAC			
hNcad-5	ACAGTGGCCACCTACAAAGG	201	55	[9]
hNcad-3	CCGAGATGGGGTTGATAATG			
E-Cadherin F	TGGAGGAATTCTTGCTTTGC	488	55	
E-Cadherin R	CGTACATGTCAGCCAGCTTC			

[a] For qPCR analysis. GAPDH F/R were used for both RT-PCR and qPCR analyses

[b] β-catenin exon 3/5 analysis

[c] http://stemcells.nih.gov/research/nihresearch/scunit/pages/RTPCR.aspx

[d] References

1. Aasen T, Raya A, Barrero MJ, Garreta E, Consiglio A, Gonzalez F, Vassena R, Bilic J, Pekarik V, Tiscornia G *et al*: **Efficient and rapid generation of induced pluripotent stem cells from human keratinocytes**. *Nat Biotechnol* 2008, **26**(11):1276-1284.
2. Brantjes H, Roose J, van De Wetering M, Clevers H: **All Tcf HMG box transcription factors interact with Groucho-related co-repressors**. *Nucleic Acids Res* 2001, **29**(7):1410-1419.
3. Takahashi K, Tanabe K, Ohnuki M, Narita M, Ichisaka T, Tomoda K, Yamanaka S: **Induction of pluripotent stem cells from adult human fibroblasts by defined factors**. *Cell* 2007, **131**(5):861-872.
4. Schostak M, Krause H, Miller K, Schrader M, Weikert S, Christoph F, Kempkensteffen C, Kollermann J: **Quantitative real-time RT-PCR of CD24 mRNA in the detection of prostate cancer**. *BMC Urol* 2006, **6**:7.

5. Surace C, Arpicco S, Dufay-Wojcicki A, Marsaud V, Bouclier C, Clay D, Cattel L, Renoir JM, Fattal E: **Lipoplexes targeting the CD44 hyaluronic acid receptor for efficient transfection of breast cancer cells**. *Mol Pharm* 2009, **6**(4):1062-1073.
6. Lung HL, Bangarusamy DK, Xie D, Cheung AK, Cheng Y, Kumaran MK, Miller L, Liu ET, Guan XY, Sham JS *et al*: **THY1 is a candidate tumour suppressor gene with decreased expression in metastatic nasopharyngeal carcinoma**. *Oncogene* 2005, **24**(43):6525-6532.
7. Nakamura K, Iinuma H, Aoyagi Y, Shibuya H, Watanabe T: **Predictive value of cancer stem-like cells and cancer-associated genetic markers for peritoneal recurrence of colorectal cancer in patients after curative surgery**. *Oncology* 2010, **78**(5-6):309-315.
8. Lee YJ, Han HJ: **Troglitazone ameliorates high glucose-induced EMT and dysfunction of SGLTs through PI3K/Akt, GSK-3 beta, Snail1, and beta-catenin in renal proximal tubule cells**. *American Journal of Physiology-Renal Physiology* 2010, **298**(5):F1263-F1275.
9. Mani SA, Guo W, Liao MJ, Eaton EN, Ayyanan A, Zhou AY, Brooks M, Reinhard F, Zhang CC, Shipitsin M *et al*: **The epithelial-mesenchymal transition generates cells with properties of stem cells**. *Cell* 2008, **133**(4):704-715.

Additional file 3: Table S3. Antibodies used

Antibody	Concentration	Catalog #	Company
β-catenin	1:1000 [a]	9587	Cell Signaling
Oct4	1:1000	2750	Cell Signaling
Nanog	1:1000	4903	Cell Signaling
Axin2	1:1000	2151	Cell Signaling
p53	1:1000	2527	Cell Signaling
Klf4	1;1000	4038	Cell Signaling
Sox2	1:1000	3579	Cell Signaling
E-cadherin	1:1000 [a]	610181	BD Pharmingen
N-cadherin	1:1000	ab18203	Abcam
CyclinD1	1:50	SC-753	Santa Cruz Biotechnology
Zeb1	1:500	3396	Cell Signaling
Fn1	1:1000	555867	BD Pharmingen
α-tubulin	1:5000	CP06	Calbiochem

[a] For IHC staining, the concentration is 1:50
